# Antibody-Based and Cell Therapies for Advanced Mastocytosis: Established and Novel Concepts

**DOI:** 10.3390/ijms242015125

**Published:** 2023-10-12

**Authors:** Peter Valent, Cem Akin, Michel Arock, Karoline V. Gleixner, Hildegard Greinix, Olivier Hermine, Hans-Peter Horny, Daniel Ivanov, Alberto Orfao, Werner Rabitsch, Andreas Reiter, Axel Schulenburg, Karl Sotlar, Wolfgang R. Sperr, Celalettin Ustun

**Affiliations:** 1 Department of Internal Medicine I, Division of Hematology and Hemostaseology, Medical University of Vienna, 1090 Vienna, Austria; 2 Ludwig Boltzmann Institute for Hematology and Oncology, Medical University of Vienna, 1090 Vienna, Austria; 3 Division of Allergy and Clinical Immunology, University of Michigan, Ann Arbor, MI 48106, USA; 4 Department of Hematological Biology, Pitié-Salpêtrière Hospital, Sorbonne University, 75013 Paris, France; 5 Division of Hematology, Medical University of Graz, 8010 Graz, Austria; 6 Service d’Hématologie, Imagine Institute Université de Paris, INSERM U1163, Centre National de Référence des Mastocytoses, Hôpital Necker, Assistance Publique Hôpitaux de Paris, 75015 Paris, France; 7 Institute of Pathology, Ludwig-Maximilians University, 80539 Munich, Germany; 8 Servicio Central de Citometria, Centro de Investigacion del Cancer (IBMCC; CSIC/USAL) Instituto Biosanitario de Salamanca (IBSAL), CIBERONC and Department of Medicine, University of Salamanca, 37007 Salamanca, Spain; 9 Department of Internal Medicine I, Stem Cell Transplantation Unit, Medical University of Vienna, 1090 Vienna, Austria; 10 Department of Hematology and Oncology, University Hospital Mannheim, 68135 Mannheim, Germany; 11 Institute of Pathology, University Hospital Salzburg, Paracelsus Medical University, 5020 Salzburg, Austria; 12 Department of Medicine, Division of Hematology, Oncology, and Cell Therapy, Coleman Foundation Blood and Marrow Transplant Center at Rush University Medical Center, Chicago, IL 60612, USA

**Keywords:** mastocytosis, stem cell transplantation, CAR-T, CAR-therapy, immunotherapy, mast cell leukemia

## Abstract

Advanced systemic mastocytosis (SM) is a heterogeneous group of myeloid neoplasms characterized by an uncontrolled expansion of mast cells (MC) in one or more internal organs, SM-induced tissue damage, and poor prognosis. Advanced SM can be categorized into aggressive SM (ASM), MC leukemia (MCL), and SM with an associated hematologic neoplasm (SM–AHN). In a vast majority of all patients, neoplastic cells display a *KIT* mutation, mostly D816V and rarely other *KIT* variants. Additional mutations in other target genes, such as *SRSF2*, *ASXL1*, or *RUNX1*, may also be identified, especially when an AHN is present. During the past 10 years, improved treatment approaches have led to a better quality of life and survival in patients with advanced SM. However, despite the availability of novel potent inhibitors of KIT D816V, not all patients enter remission and others relapse, often with a multi-mutated and sometimes *KIT* D816V-negative disease exhibiting multi-drug resistance. For these patients, (poly)chemotherapy, antibody-based therapies, and allogeneic hematopoietic stem cell transplantation may be viable treatment alternatives. In this article, we discuss treatment options for patients with drug-resistant advanced SM, including novel KIT-targeting drugs, antibody-based drugs, and stem cell-eradicating therapies.

## 1. Introduction

Systemic mastocytosis (SM) is a group of stem cell-derived, hematopoietic neoplasms characterized by uncontrolled expansion and accumulation of tissue mast cells (MC) in various internal organs, including the bone marrow (BM), spleen, liver, and gastrointestinal (GI) tract [1,2,3]. In a subset of patients, the skin may be involved [4]. However, unlike patients with typical indolent SM, some patients with advanced SM may only have a few or no skin lesions. In patients with advanced SM, MC infiltration in various organs is associated with organ damage [1,2,3]. Due to improved diagnosis, the prevalence of (advanced) SM has increased over the past few years. The estimated prevalence of adult SM is approximately 1 per 10,000 in the Western world.

According to the classification of the World Health Organization (WHO), advanced SM can be categorized into aggressive SM (ASM), SM with an associated hematologic neoplasm (SM–AHN), and MC leukemia (MCL) [1,2,3,5,6,7,8,9]. ASM can further be split into a non-transformed variant and ASM in transformation to MCL (ASM-t) where MC in BM smears account for 5–19% of all nucleated cells [8,10]. SM–AHN is further divided into patients with indolent SM (ISM–AHN), ASM–AHN, and MCL–AHN [8,10]. Furthermore, SM–AHN is also classified based on the type of AHN [1,5,6,7,8].

MCL can be divided into a leukemic variant (MC in peripheral blood ≥ 10%) and an aleukemic variant (MC in blood < 10%) [5,6,7,8,10]. In addition, MCL is split into a primary form of MCL (de novo MCL) and secondary MCL arising from another form of mastocytosis, such as smoldering SM (SSM), ASM, SM–AHN, or MC sarcoma (MCS) [8,10]. Especially in ASM-t and MCS, the rate of transition into MCL is high [10,11,12,13]. Therefore, although not an SM as per SM criteria, MCS is a high-grade MC neoplasm where cytoreductive therapy and other intensive treatment approaches have to be considered.

In most patients with advanced SM, neoplastic cells display transforming mutations in the *KIT* oncogene (Table 1) [14,15,16,17]. These mutations lead to cytokine-independent autonomous growth of neoplastic MC progenitors. The most prevalent mutant form of *KIT* is D816V. This mutation is found in all categories of advanced SM, and often also in the AHN portion of the disease. However, in some patients with ASM, MCL, or MCS, other KIT-activating mutations may be detected [11,12,13,14,15,16,17]. The same holds true for pediatric SM, familial SM, and the well-differentiated subtypes of SM. Moreover, in patients with advanced SM, especially SM–AHN, neoplastic cells often exhibit additional mutations in clinically relevant genes [15,16,17,18,19,20,21,22,23]. These include mutations in *SRSF2*, *ASXL1*, *RUNX1*, or *DNMT3A*, which may be associated with disease evolution and progression as well as drug resistance in neoplastic cells [19,20,21,22]. Sometimes, more malignant subclones, especially AHN-related clones that are drug resistant and lack *KIT* D816V, develop (through selection) in these patients, even if the initial dominant sub-clone displayed *KIT* D816V.

Another major problem in SM is MC activation. In fact, these patients often suffer from recurrent, mediator-induced symptoms or even anaphylaxis, especially when a concomitant, IgE-dependent allergy is also present [3,4,5,6,7,8]. Mediator-related symptoms include, among others, flushing, itching, cramping, diarrhea, and hypotension [3,4,5,6,7,8]. In many cases, allergens are inducing these symptoms. However, patients with indolent or advanced SM may also develop these symptoms upon drug exposure.

## 2. Standard Treatment Options for Patients with Advanced SM

In the past 10 years, the treatment of patients with advanced SM has improved substantially. First, a number of KIT D816V-targeting tyrosine kinase inhibitors (TKI) have been developed and have been applied successfully in patients with advanced SM [24,25,26,27,28,29,30,31,32,33]. Two of these TKI have recently been approved for the treatment of advanced (resistant) SM by major health authorities in the US and EU, namely midostaurin and avapritinib. Both agents are able to suppress disease-related symptoms and the expansion of neoplastic MC in a considerable number of patients [26,27,28,29,30,31,32,33]. Avapritinib is a superior drug that induces hematologic remission in a substantial subset of patients with advanced SM [30,31,32,33]. Based on their clinical efficacy, midostaurin and avapritinib are considered first-line agents in the treatment of advanced SM. However, despite impressive results, some patients continue to progress or relapse, often in the form of a *KIT* D816V-negative disease [30,31,32,33]. For such cases, alternative treatment options, including experimental drugs, have to be considered. Apart from KIT D816V-targeting drugs, also other anti-neoplastic agents are available for patients with advanced SM. Some of these patients may respond to cladribine (2CdA) [34,35,36,37,38]. In other patients, especially those with rapid progression to MCL or another (acute) leukemia, poly-chemotherapy or even allogeneic hematopoietic stem cell transplantation (alloHSCT, referred to as HSCT in this paper) are recommended [1,2,6]. Hydroxyurea is commonly used as a palliative drug in advanced SM. Table 2 provides a summary of treatment options for patients with advanced SM.

**Figure 1:**
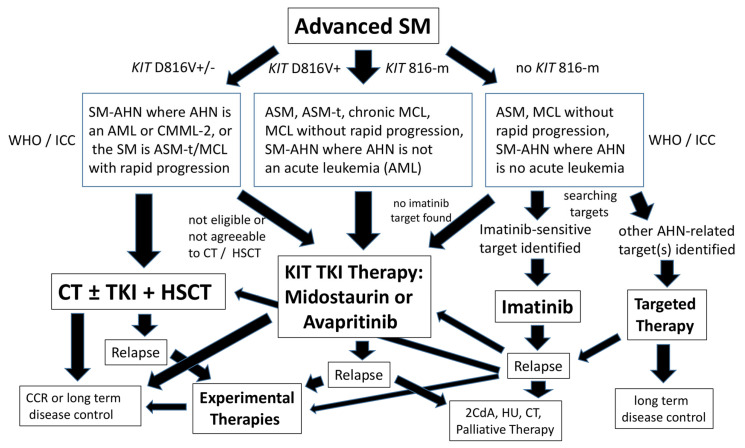
Treatment algorithm for patients with advanced systemic mastocytosis (SM). After having established the diagnosis of advanced SM, the variant of disease, the clinical behavior (aggressive with rapid progression or indolent), and the presence and type of AHN need to be determined using WHO criteria and/or criteria provided by the international consensus classification (ICC). In addition, the presence and type of molecular targets need to be defined. In patients with *KIT* D816V+ advanced SM or other similar mutations at codon 816 (KIT 816-m), tyrosine kinase inhibitors (TKI) directed against KIT D816V are usually applied as first-line therapy. Two such TKI have been approved by the FDA and EMA: midostaurin and avapritinib. In patients who have a rapidly progressing ASM or MCL or SM–AHN where the AHN is an acute myeloid leukemia (AML) or rapidly progressing chronic myelomonocytic leukemia (CMML), intensive poly-chemotherapy (CT) should be considered as an alternative first-line treatment option. In patients who are eligible and respond to TKI or CT, subsequent allogeneic hematopoietic stem cell transplantation should then also be considered. In addition, patients who relapse after drug therapy should be considered for CT and subsequent HSCT. However, such patients may also be candidates for experimental drugs or palliative drugs. In patients with advanced SM who have no *KIT* mutation or a mutant form sensitive to imatinib, imatinib may be considered as first line therapy. Finally, in patients with SM–AHN in whom the AHN is an aggressive disease exhibiting certain molecular targets, targeted drug therapies are usually recommended. The standard palliative drug for patients with drug-resistant disease who relapsed after CT or HSCT, remains hydroxyurea (HU). Abbreviations: SM, systemic mastocytosis; ASM, aggressive SM; SM–AHN, SM with an associated hematologic neoplasm; MCL, mast cell leukemia; CCR, continuous complete remission; WHO, world health organization; FDA, Food and Drug Administration; EMA; European Medicines Agency.

When considering treatment options in a patient with advanced SM, the first important questions are whether the disease is rapidly progressing, whether an AHN is present, and whether most or all disease components exhibit the *KIT* D816V mutation (Figure 1). In addition, it is important to know whether the patient is eligible for intensive therapy (Figure 1). In those with *KIT* D816V-positive advanced SM, KIT D816V-targeting drugs (midostaurin or avapritinib) are usually recommended as first-line therapy [1,2,25,26,27,28,29,30,31,32,33]. When TKI are not available or the patient is intolerant, cladribine (2CdA) may be considered. However, although 2CdA is effective, response rates are lower compared to those seen with midostaurin or avapritinib [34,35,36,37,38]. In patients who have *KIT* D816V-negative advanced SM, other KIT-targeting TKI, such as imatinib, may be considered, especially when *KIT* sequencing reveals a sensitive mutation or wild-type *KIT* (Figure 1, Table 2) [39,40,41,42]. Midostaurin or avapritinib may also be applied in such patients (Figure 1). It is important to note in this regard that in about 80% of patients with well-differentiated advanced SM, in about 20–30% of all cases with MCL, and in about 90% of all patients with true (primary) MCS, neoplastic cells lack *KIT* D816V (Table 1). In several of the patients with MCL, MC display other mutations in codon 816, such as D816H or D816Y. It is also important to note that testing for the *KIT* D816V mutation should be performed with a highly sensitive PCR test in order to avoid false-negative results. In patients with well-differentiated SM, a KIT mutant form that is responsive to imatinib, may be detected [39,40,41,42,43]. In most patients with true MCS, no *KIT* mutations are found, and neoplastic cells are usually resistant against KIT-targeting and conventional drugs, including chemotherapy [11,12,13].

For patients with rapidly progressing ASM or MCL, poly-chemotherapy with or without subsequent HSCT is often recommended (Figure 1) [1,2,44,45,46,47]. For those who are eligible for HSCT, the optimal way to proceed may be to start debulking (by a TKI and/or chemotherapy) and to introduce HSCT as early as possible [44,45,46]. This also holds true for patients with MCS, as many of these cases transform to MCL [11,12,13,48].

## 3. Special Considerations for the Treatment of Patients with SM–AHN

In patients with SM–AHN, treatment of the AHN is often required. Indeed, in such cases, the AHN may be an aggressive malignancy. In these patients, it is essential to know whether most AHN cells (subclones) carry *KIT* D816V. Likewise, in SM with chronic myelomonocytic leukemia (SM–CMML), neoplastic monocytes are often *KIT* D816V-positive, which may favor treatment with a KIT D816V-targeting drug [49,50]. By contrast, in acute myeloid leukemia (AML), some or even most AML subclones may lack *KIT* D816V, so that treatment with a KIT D816V-targeting drug alone may even lead to selection of more KIT D816V-negative sub-clones [51,52,53]. In general, patients with SM–AHN should be treated for their AHN as if no SM was diagnosed, and the SM portion of the disease should be treated as if no AHN was found [1,2,3,5,6,7,45,47]. This strategy implies that several of these patients receive combinations of anti-neoplastic drugs or sequential drug therapies. For example, in a patient with ASM–AML exhibiting *KIT* D816V and a *FLT3*-ITD mutation, the disease may be treated with midostaurin, together with chemotherapy (direct combination). Thus, it is important that both the SM and the AHN portion of the disease are treated in a target-specific manner. Therefore, it is also of crucial importance to establish the correct final diagnosis in each case, and to define both disease components by adding the molecular signature in the report [1,2,3,5,6,7,45,47]. For example, in a patient with ISM expressing *KIT* D816V with an associated *JAK2* V617F-positive primary myelofibrosis (PMF), the final diagnosis is ISM–PMF exhibiting *KIT* D816V (in SM cells) and *JAK2* V617F (in MPN cells). In such patient, no KIT D816V-targeting drug is required, but when PMF-related symptoms occur, the patient may be a candidate for treatment with a JAK2 V617F-targeting drug.

In some patients with SM–AHN, it may be difficult to interpret potential C-findings (like cytopenia), especially when both the SM portion and the AHN could cause cytopenia, for example, ASM with associated myelodysplastic syndromes/neoplasm (MDS). In these patients, the AHN may be an advanced *KIT* D816V-negative MDS (or secondary AML) requiring treatment with 5-azacitidine (AZA) or AZA plus venetoclax. In such cases, the AHN portion of the disease may or may not respond to these standard treatments in the same way as in patients without SM [54,55,56]. Midostaurin and avapritinib may also be effective in these cases, especially when most or all AHN cells are *KIT* mutated, or are driven by KIT-induced oncogenic pathways [57,58]. However, in SM–AML, some or even most AML-subclones may lack *KIT* D816V [51,52]. The same holds true for patients with MCS and some with MCL. Overall, it seems as if more advanced, progressive SM is associated with the development of multiple drug-resistant subclones, and some or even most of these subclones lack KIT mutant forms including *KIT* D816V.

In patients in whom the *KIT* D816V mutation status is unknown or no *KIT* mutation was found, a KIT inhibitor may still be a therapeutic option. Indeed, midostaurin and avapritinib suppress the activity of wild-type KIT, and both may exert anti-neoplastic effects in SM regardless of the *KIT* mutation status. Sometimes, *KIT* D816V is not detected in neoplastic cells based on the low sensitivity of the assay. Therefore, we recommend testing for *KIT* D816V with a highly sensitive PCR assay before starting therapy in all patients [15,16,17,59].

As mentioned, when both the SM and the AHN require specific anti-neoplastic therapies, drug combinations have to be considered. In patients with an aggressive malignancy, such as MCL and/or AML, intensive therapy (plus TKI therapy) is introduced to achieve remission before the patient is prepared for allogeneic HSCT [1,2,45,46,47,58]. In those who are not eligible for HSCT, continuous treatment with avapritinib may be an alternative treatment option.

## 4. Treatment Options for Patients with Drug-Resistant Advanced SM

The prognosis for patients with advanced SM who are resistant against KIT-targeting drugs and/or other anti-neoplastic drugs, including 2CdA, is usually dismal. Some of these patients may not have yet been treated with avapritinib because the drug was not available. These patients may benefit from avapritinib therapy [32,33]. Other patients may benefit from experimental drugs or drug combinations using TKI (midostaurin, avapritinib) and other targeted drugs such as venetoclax, or TKI and conventional antineoplastic drugs such as 2CdA. However, most responses are only transient and are followed by a relapse. Therefore, when eligible, these patients are prepared for HSCT. If HSCT is not a viable option, patients are treated with experimental chemotherapy or palliative drugs such as hydroxyurea (HU) [1,2,45]. In those with drug-resistant SM–AML who are not eligible for HSCT, chemotherapy regimens containing AZA, 2CdA, venetoclax, or other drugs may be offered [1,2,45,60]. However, most of these patients have a short survival time.

## 5. Allogeneic HSCT

HSCT remains the only potentially curative treatment for patients with advanced SM who are resistant against multiple drug therapies. Especially young and fit patients with multi-resistant advanced SM may benefit from HSCT [44,45,46,56,58,61,62,63,64]. In these patients, graft versus mastocytosis effects have been documented [44,62,63]. However, so far, no controlled clinical studies exploring the definitive value of HSCT in patients with advanced, drug-resistant SM have been performed. In one study, patients with advanced SM who were treated with HSCT (either first-line or after drug therapy) were examined in a retrospective multi-center study [44]. In this, study, patients with ASM, MCL, and SM–AHN were included. The outcomes after HSCT concerning survival and relapse-free survival were found to be better for those who had ASM or SM–AHN compared to those with MCL [44]. The outcomes after HSCT were also found to be more favorable when myeloablative conditioning was performed compared to dose-reduced (non-myeloablative) conditioning [44]. There are additional factors that may impact on outcome and prognosis of HSCT in patients with advanced SM. For example, the outcome may be better when successful debulking can be performed prior to HSCT. Indeed, in patients in whom debulking with targeted drugs or chemotherapy failed, HSCT is difficult to perform, and post-HSCT outcomes are usually poor. Therefore, most experts recommend early HSCT and early pre-HSCT therapy in eligible patients with advanced SM. The type of debulking therapy depends on the type of AHN, the presence of *KIT* D816V, and other molecular targets displayed by neoplastic cells. In those with a high variant allele frequency of *KIT* D816V, avapritinib may be appropriate and sufficient to induce debulking [58]. In the case of SM–AML, poly-chemotherapy should be considered as the most effective and most promising approach to achieve substantial debulking before HSCT.

There is also a growing discussion about the use of KIT-targeting drugs (midostaurin, avapritinib) after successful HSCT. Indeed, based on case report observations, such treatment, when introduced after hematologic regeneration following HSCT (for example from day +100), may keep the neoplastic disease process under control [28,64]. However, no results from controlled clinical trials are available, and it remains unknown whether such post-HSCT therapy exerts major effects on residual neoplastic stem cells (NSC) as a useful maintenance therapy. In addition, it remains uncertain how long such post-HSCT therapy with KIT-targeting drugs should be performed. In most centers, such patients have been treated with midostaurin for 1 or 2 years post-HSCT. In patients with measurable minimal residual disease (by *KIT* D816V PCR), longer treatment with KIT-targeting drugs may be considered. Avapritinib has recently been applied post-HSCT in patients with *RUNX1-RUNX1T1*-positive *KIT*-mutated AML with minimal residual disease who failed immunotherapy with interferon alpha or donor lymphocyte infusions [65]. In these patients, cytopenia occurred frequently. However, no clinical studies using avapritinib post-HSCT in advanced SM have been conducted to date.

## 6. Antibody-Based Treatment of Drug-Resistant Advanced SM

Although antibody-based drug therapies are available for patients with high-risk AML and therapy-refractory AML, no antibody-based therapy for patients with MCL or other forms of advanced SM is available. Since most MC and most NSC, including AHN-related NSC, display CD33 in most SM patients (Table 3) [66,67,68,69], the CD33-targeted drug conjugate gemtuzumab ozogamicin (GO) has been tested in preclinical studies. Based on its anti-neoplastic effects on neoplastic MC and NSC in in vitro studies [67,68], GO has been proposed for the treatment of advanced SM. However, only a few anecdotal reports have been published so far. In one report, treatment of a patient with drug-resistant SM–AHN resulted in disease debulking and sustained remission [70]. In other patients with advanced SM, treatment with GO and poly-chemotherapy resulted in a substantial decrease in the numbers of NSC [68]. There are also other surface targets that are expressed on neoplastic MC and/or NSC, and may therefore serve as molecular targets in advanced SM (see below).

## 7. Target Expression Profiles of NSC in Advanced SM

**Figure 2:**
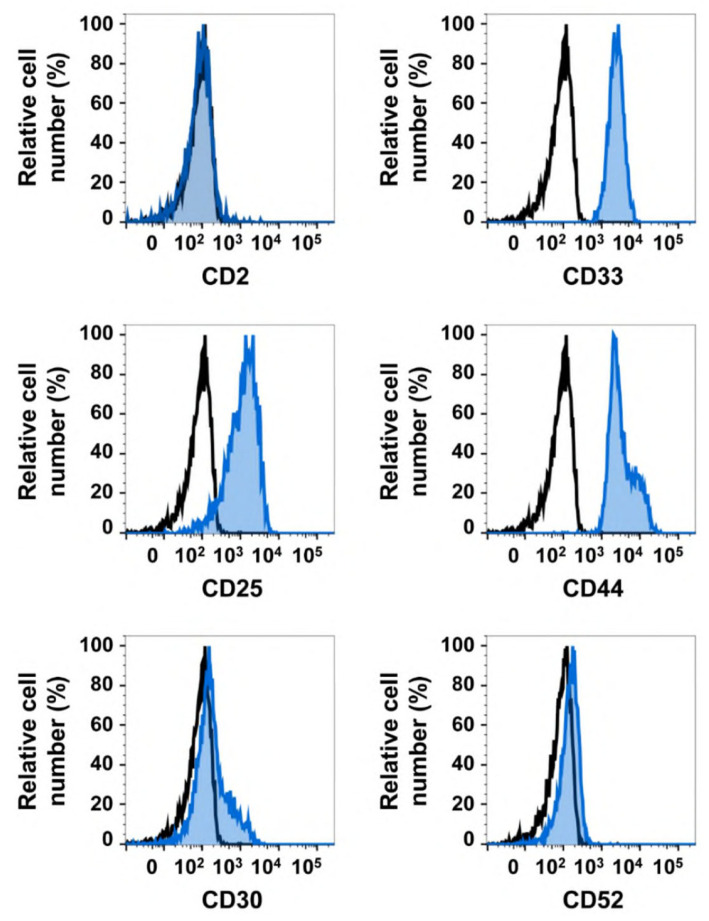
Expressions of cell surface markers on neoplastic stem cells in SM. Bone marrow mononuclear cells were obtained from a patient with systemic mastocytosis (SM) with an associated hematologic neoplasm (SM–AHN), and stained with phycoerythrin (PE)-conjugated monoclonal antibodies directed against CD2, CD25, CD30, CD33, CD44, and CD52, as well as fluorochrome-labeled antibodies against CD34 and CD38. Expressions of target antigens on CD34^+^/CD38^−^ stem cells were assessed via multi-color flow cytometry, and are indicated by the blue histograms. The isotype-matched control antibody is also shown (black open histograms). Representative histograms show expressions of CD25, CD33, and CD44 on neoplastic stem cells, whereas these cells expressed only trace amounts of CD30 and CD52, and stained negative for CD2. Stem cells also displayed CD123 (not shown), confirming the neoplastic nature of these cells.

Recent data suggest that NSC in advanced SM (including ASM, SM–AHN, and MCL) reside in a small CD34^+^/CD38^−^ fraction of the malignant clone [68]. These cells have a selective potential to initiate and propagate the disease in vivo in NSG mice exhibiting human membrane-bound stem cell factor (NSG_hSCF_ mice) [68]. Although not all observations can be translated from mouse models to the human system, these NSC may also propagate the disease in patients with advanced SM for unlimited time periods. By contrast, the more mature cells in the same disease, including CD34^+^/CD38^+^ progenitor cells and the bulk of neoplastic MC, are unable to initiate and propagate the malignancy in vivo [68]. As a consequence, any therapy can only act as a curative approach when eliminating most or all CD34^+^/CD38^−^ NSC in a given patient. In most patients with advanced SM, CD34^+^/CD38^−^ NSC exhibit the key stem cell markers CD13 (aminopeptidase-N), CD123 (IL-3 receptor alpha), and CD133 (AC133) [68]. These antigens are also expressed on neoplastic MC in SM, and often also on AHN cells. In addition, NSC as well as MC express a number of cell surface targets, such as CD33 (Siglec-3), CD44 (Hermes), and CD117 (KIT) (Figure 2, Table 3) [67,68,69,70,71,72,73,74,75]. In contrast, other immunological targets, such as CD30 (Ki-1) or CD327 (Siglec-6), are only detectable on neoplastic MC, but are not detectable (or only found in trace amounts) on NSC in advanced SM (Table 3) [76,77,78]. These targets may be less attractive, since therapies directed against these antigens would only lead to an eradication of MC, but not to an elimination of NSC. Another important aspect is that almost all surface targets identified on NSC in advanced SM are also expressed on normal hematopoietic stem cells. This holds true for CD33, CD44, CD117, and CD123. As a consequence, targeted treatment approaches can lead to the eradication of normal hematopoietic stem cells, and thus prolonged cytopenia. This is a major issue when considering the development of antibody-based therapies or cell-based therapies such as CAR-T or CAR-NK cell therapies. On the other hand, several of these targets are expressed on NSC at much higher levels compared to normal stem cells, so that treatment with antibody-based targeted drugs may be a feasible approach because of the therapeutic window. This may hold true for CD33, CD44, and CD123. Indeed, the treatment of AML with the CD33-based toxin conjugate GO at recommended doses is often associated with prolonged cytopenia, but usually does not lead to irreversible aplasia [79]. Whether this is also the case with antibody-based drugs directed against CD117 or CD123 remains unknown. For example, no useful therapeutic window has been identified for CD117 on neoplastic MC or NSC in SM, as the surface expression is sometimes even lower on NSC compared to normal stem cells [68]. However, there is still some hope that CD117-targeted drug therapies may not lead to complete stem cell exhaustion. In fact, in patients with Ph+ chronic myeloid leukemia, long-term treatment with strong inhibitors of KIT such as imatinib does not lead to severe aplasia, even when the patients are treated for several decades [80]. Finally, some of the cell-based therapies, such as CAR-T cell therapies, may be combined with HSCT in order to solve the problem of eradication of normal stem cells.

## 8. Novel Approaches to Target NSC in Advanced SM

A number of therapeutic approaches directed against certain surface antigens (targets) expressed on NSC have been developed. Antibody-based therapies include antibody–toxin conjugates, bi-specific or tri-specific linker-constructs, or antibodies that kill target cells through complement-dependent mechanisms [68,77,81]. Whereas the antibody–toxin conjugate GO has been applied in a few cases, bi-specific or tri-specific linker-constructs have so far not been applied in clinical practice in patients with advanced SM. Recently, however, a tri-specific killer engager CD16xIL15xCD33 that induces NK cell activation and cytotoxicity against neoplastic MC has been presented [81]. Whether such engager constructs will be able to eliminate neoplastic MC and NSC in patients with advanced SM remains at present unknown. In addition, it remains unknown whether CAR-T cell or CAR-NK cell therapies will be developed far enough to reach clinical application in interventional trials. Currently, major efforts are being undertaken to establish CAR-T cell approaches directed against CD33, KIT, and a few other targets, with the aim to eliminate NSC in patients with advanced SM, including SM–AHN. One specific aspect to consider with these therapies is that antibodies and CAR cells (CAR-T or CAR-NK) attacking MC may not only induce a tumor lysis syndrome, but also a MC mediator release syndrome, which may result in severe life-threatening anaphylaxis, especially when MC are destroyed rapidly, so that an excess amount of mediators and cytokines are released.

## 9. Concluding Remarks and Future Perspectives

Despite the availability of novel strong TKI and other effective conventional drugs, advanced SM is still a major challenge in clinical hematology, and many patients die from drug-resistant disease. One major issue in advanced SM is that the disease-initiating and propagating NSC exhibit multiple forms of drug resistance. A potent approach to overcoming the resistance is to attack these cells directly with cell-based therapies and antibody-based therapies. Indeed, HSCT is still the only potentially curative treatment approach for patients with drug-resistant advanced SM. In these patients, pre-transplant debulking is usually performed, often by administering (poly)chemotherapy and/or novel KIT-targeting drugs. In addition, antibodies directed against NSC such as GO may be considered, especially when a concomitant AML is also detected. Finally, post-HSCT treatment with KIT-targeting drugs may be considered in future algorithms, with the aim to suppress minimal residual disease (residual NSC) and to maintain disease-free survival. This may be especially important for patients who have multi-drug-resistant KIT D816V+ ASM or MCL. In summary, NSC-targeting therapies, including HSCT and antibody-based approaches, are essential weapons to treat patients with drug-resistant advanced SM. There is also hope that novel therapies directed against NSC will further improve survival in advanced SM in the foreseeable future.

## Figures and Tables

**Table 1:** Estimated prevalence of *KIT* D816V, other *KIT* mutations, and mutations in other genes in advanced mast cell neoplasms.

Disease Variant	Prevalence of *KIT* D816V	Other *KIT* Mutations	Other Gene Variants *
SSM	>90%	<10%	<10%
SM–AHN	>90%	<10%	>70%
ASM	80–90%	10–20% **	10–20%
AdvSM_WD_	20%	50–80% **	<10%
MCL	70–80%	20–30% **	10%
MCS ***	<10% ***	<10%	10–30% ***

* Other genetic abnormalities (apart from *KIT* mutations) are often detected in patients with SM with associated hematologic neoplasm (SM–AHN) and include, among others, mutations in *RUNX1*, *TET2*, *SRSF2*, *RAS*, or *ASXL1*. ** Some of the resulting KIT mutant forms are sensitive against imatinib, whereas KIT D816V confers resistance against imatinib. *** In true (primary) MCS without features of SM, no *KIT* D816V or other *KIT* mutations are found. Abbreviations: SM, systemic mastocytosis; SSM, smoldering SM; ASM, aggressive SM; AdvSM, advanced SM; SM_WD_, well-differentiated SM; MCL, mast cell leukemia; MCS, mast cell sarcoma.

**Table 2:** Treatment options for patients with advanced mastocytosis.

Treatment	Indications
Interferon-alpha (IFN-A) *	Treatment-refractory osteoporosis (low dose IFN-A). ASM with liver involvement and ascites (patient not eligible for TKI therapy or 2CdA). IFN-A responsive AHN.
Cladribine (2CdA)	Patient not eligible for KIT TKI therapy or patient resistant against KIT TKI or no KIT TKI available.
Imatinib	*KIT* D816V-negative advanced SM, including ASM with *KIT* K509I.
Masitinib	*KIT* D816V-negative advanced SM.
Midostaurin	ASM, ASM-t, ASM–AHN, MCL, MCL–AHN. ISM–AHN where the AHN is a KIT-driven (*KIT* D816V+) aggressive neoplasm.
Avapritinib	ASM, ASM-t, ASM–AHN, MCL, MCL–AHN. ISM–AHN where the AHN is a KIT-driven (*KIT* D816V+) aggressive neoplasm.
Hydroxyurea (HU)	Multi-drug-resistant advanced SM, including ASM, MCL and SM–AHN. HU is a standard palliative drug.
Local radiation	Mast cell sarcoma (MCS) or MCS-like progression of ASM. Huge splenomegaly: as debulking prior to CT. Skeletal disease (huge osteolysis with local tumor mass).
Poly-chemotherapy	Drug-resistant advanced SM. Debulking as preparation for HSCT. SM–CMML with progressing CMML or SM–AML in patients not eligible for HSCT.
Mono-chemotherapy: demethylating agents and other drugs, such as venetoclax	Patients with advanced, KIT TKI-resistant SM not eligible for poly-chemotherapy or HSCT (and/or resistant against 2CdA). SM–AHN patients not eligible for HSCT in whom the AHN may be responsive: (example: azacitidine in MDS or AML).
Allogeneic HSCT	Drug resistant advanced SM, ASM-t or MCL. ASM with rapid progression. SM–CMML, SM–AML.

* During the initial few weeks of IFN-A, the drug is often applied together with oral prednisolone (starting at 1 mg/kg/day). Abbreviations: TKI, tyrosine kinase inhibitor; AHN, associated hematologic neoplasm; SM, systemic mastocytosis; ASM, aggressive SM; ASM-t, ASM in transformation; MCL, mast cell leukemia; MDS, myelodysplastic syndrome; ISM, indolent SM; CT, poly-chemotherapy; HSCT, (allogeneic) hematopoietic stem cell transplantation; CMML, chronic myelomonocytic leukemia; AML, acute myeloid leukemia.

**Table 3:** Cell surface target expression profiles of neoplastic mast cells and neoplastic stem cells in patients with advanced SM.

CD	Antigen, Target	Cell Surface Expression Detected by Flow Cytometry * on					
		Neoplastic Mast Cells			CD34^+^/CD38^−^ Stem Cells		
		ASM	SM–AHN	MCL	ASM	SM–AHN	MCL
CD2	LFA-2	+	+	+/−	−	−	−
CD13	A-Pept-N	+	+	+	+	+	+
CD25	IL-2RA	+	+	+	+/−	+/-	+/-
CD30	Ki-1	+/−	+/−	+/−	−/+	−/+	−/+
CD33	Siglec-3	+	+	+	+	+	+
CD44	Hermes	+	+	+	+	+	+
CD47	IAP	+	+	+	+	+	+
CD52	Campath-1	+	+	+	−/+	−/+	−/+
CD117	KIT	+	+	+	+	+	+
CD123	IL-3RA	+/−	+/−	+/−	+	+	+
CD184	CXCR4	+	+	+	+	+	+
CD274	PD-L1	+	+	+	n.k.	n.k.	n.k.
CD327	Siglec-6	+	+	+	−	−	−

* Surface expressions of targets were analyzed with fluorochrome-labeled antibodies and multi-color flow cytometry. Expressions of targets on KIT^++^ mast cells and CD34^+^/CD38^−^ stem cells were quantified using the following score: +, clearly expressed on >75% of all cells; +/−, expressed weakly or only on a subset (10–50%) of all cells; −/+, expressed only in trace amounts or in a very small sub-fraction of cells (<10%). Abbreviations: SM, systemic mastocytosis; ASM, aggressive SM; SM–AHN, SM with an associated hematologic neoplasm; MCL, mast cell leukemia; A-Pept-N, aminopeptidase N; IL-2RA, interleukin-2 receptor alpha chain; IAP-1, integrin-associated protein; IL-3RA, interleukin-3 receptor alpha chain; PD-L1, programmed death ligand-1; n.k., not known.
